# Pooling serum samples may lead to loss of potential biomarkers in SELDI-ToF MS proteomic profiling

**DOI:** 10.1186/1477-5956-6-16

**Published:** 2008-06-01

**Authors:** S Tariq Sadiq, Dan Agranoff

**Affiliations:** 1 Centre for Infection, Cellular & Molecular Medicine, St George's University of London, Cranmer Terrace, SW17, London, UK; 2 Department of Infectious Diseases and Immunity, Imperial College London (Hammersmith campus), Commonwealth Bldg, Du Cane Rd, W12 ONN, London, UK

## Abstract

**Background:**

High throughput proteomic technology offers promise for the detection of disease biomarkers and proteomic signature patterns but biomarker discovery studies can be limited by cost factors when large sample size numbers are required. Pooling sera or plasma samples from disease cases potentially offers a solution to cost implications by reducing the standard errors of mass to charge values. Surface enhanced laser desorption/ionization time of flight (SELDI-ToF) mass spectra obtained from individual and pooled sera from invasive aspergillosis cases and controls were compared.

**Results:**

Pooling resulted in 50% loss of peak clusters detected in individual samples. Overall, loss was greatest for low intensity clusters. Peak intensities and case:control intensity ratios, among clusters not lost, demonstrated good reproducibility.

**Conclusion:**

Pooling sera results in significant potential biomarker loss when using SELDI-ToF MS.

## Background

High throughput proteomic technology has increasingly been used for the detection of disease biomarkers and proteomic signature patterns have potential diagnostic value [1]. However biomarker discovery studies can be limited by cost factors when large sample size numbers are required. Surface enhanced laser desorption/ionization time of flight mass spectrometry (SELDI-ToF MS) [2] is a high-throughput proteomic profiling platform that has reasonable reproducibility if strict quality control procedures are observed [3]. Potential biomarkers in sera or tissue are often found at very low and variable concentrations and finding robust disease associations for them is a major challenge [4]. Pre-fractionation of samples by ion-exchange chromatography, coupled with further on-chip fractionation by the SELDI method, improves detection of low abundance protein species but requires larger numbers of array experiments, pushing up costs further.

One proposed way in which to address the challenge of cost is to pool patient samples during the discovery phase. In theory by pooling equal volumes of sera from cases and similarly from controls, the potential loss of statistical power arising from reduced sample size is theoretically offset by the reduction in the standard error of the means of individual protein mass to charge *(m/z) *values (peak clusters), as long as the reduction in sample size is not too marked. However it is unclear as to whether the process of pooling itself actually results in simple averaging of protein concentrations because of potential biological or biochemical interactions, although this would be less likely if samples are denatured prior to pooling. Importantly for biomarker discovery, ratios of the biomarker concentrations in cases compared to controls found in individual samples would need to be maintained when pooled sample analysis is performed.

The aim of this study was to investigate the consistency of potential biomarker detection and variation in intensities for individual peak clusters obtained from SELDI-ToF before and after pooling serum samples.

## Methods

### Generation of mass spectra

Equal volumes of sera from 20 patients with proven invasive aspergillosis (an opportunistic fungal infection affecting immunocompromised patients) and 20 controls with non-fungal causes of sepsis, were pooled to form case and control pooled samples. After denaturation for 30 minutes on ice (10 μl pooled or individual serum diluted 1:10 v/v in 9 M urea/2% CHAPS/2 mM DTT/50 mM Trizma base (pH 9.0)), samples were further diluted 1:10 v/v in binding buffer (100 mmol/L ammonium acetate, 0.1% Triton-X100, pH 4.0), and immediately spotted in duplicate on CM10 weak cation exchange arrays (Ciphergen Inc, CA), pre-equilibrated with binding buffer, in a bioprocessor (100 μL/well). The bioprocessor was sealed and incubated at room temperature on a shaker (300 rpm) for 1 hr after which the protein chip arrays were washed three times in binding buffer, twice in wash buffer (100 mmol/L ammonium acetate, pH4.0) and finally in water (5 minutes/wash). Mass spectra were generated by SELDI-ToF MS using a Proteinchip reader PBSIIc (Ciphergen Inc, CA) as described previously [5,6]. The pooled serum samples were run at the same time, on the same SELDI-ToF mass spectrometer as individual (non-pooled) samples, without any intervening period of storage and were interspersed among individual samples on the same proteinchip arrays. Spectra were generated at a laser intensity of 215, high mass 100 kDa, detector sensitivity 8, and focus mass 10 kDa (for low mass range) and 50 kDa (high mass range). Spectra were baseline-subtracted and normalised to total ion current. Samples were re-run or excluded if their normalisation factor (NF) fell outside 2 SDs from the mean NF. The instrument was calibrated for the *m/z *ranges 2500 – 20,000 and 20,000–100,000 using two pre-mixed sets of external standards; All-in-one Peptide Standard (Ciphergen In., CA) and All-in-One Protein Standard (Ciphergen Inc., CA), respectively. To assess technical reproducibility, a single quality control serum sample collected and aliquoted from 1 healthy volunteer was run repeatedly at random spot positions on 7 separate CM10 protein chip arrays on the same day as the individual and pooled samples. The pooled coefficients of variation (CV_p_) for intensity for all peaks with S/N > 5, present in 100% of replicates, were 17.3% (degrees of freedom 222) for the *m/z *range 2,500 – 20,000 (37 peaks) and 17.6% (df 48) for the *m/z *range 20,000–100,000 (8 peaks). CV_p_s for *m/z *in the same *m/z *ranges were 0.03% and 0.05%, respectively. These values are consistent with those reported in the literature and with the manufacturer's specifications.

### Peak clustering

Peak clusters are *m/z *values deemed to represent corresponding peaks across all spectra. After baseline subtraction and normalisation to total ion current, peak clusters were generated from the individual sample spectra using Biomarker Wizard (range 2,500 – 100,000, 1st pass S/N 5, 2nd pass S/N 2, cluster mass window 0.3%). As a further quality control measure, a further set of clusters was generated using the same parameters, specifically across the mass range of 20,000–100,000 but using a wider mass window of 0.6% in order to check that the sensitivity of cluster detection was not significantly affected using a mass window of 0.3% at the higher mass range. Average peak intensities were calculated from the duplicate spots.

**Figure 1:**
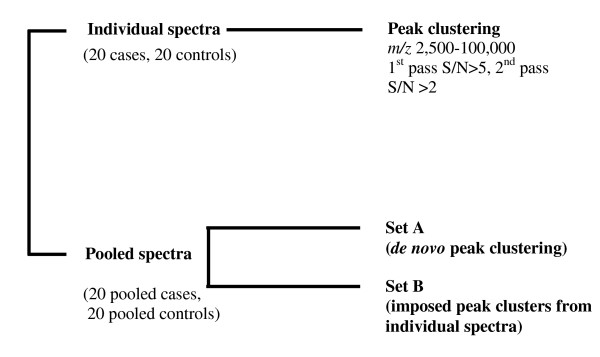
Peak-cluster sets analysed in this study.

There are 2 ways of identifying corresponding peak clusters in the individual and pooled sets of spectra. Peaks can be detected *de novo *in the pooled spectra using the same detection criteria as for individual spectra. Alternatively, the peak clusters derived from the individual spectra can be 'superimposed' on the pooled spectra to provide an intensity value for every notional peak cluster, regardless of whether it is actually represented in the pooled spectra. Because we wanted to examine the effects of pooling on the detectability and intensity of peaks, we used both approaches. 'Set A' was generated by performing *de novo *peak clustering on the pooled spectra and 'Set B' by imposing peak clusters derived from the individual spectra, as summarised in figure 1.

Because peak clusters obtained from set A did not always align precisely with probable corresponding clusters obtained from the individual spectra, it was necessary to perform a secondary clustering procedure to define corresponding peaks. Thus peak clusters from the individual and pooled samples that were closest and that fell within 0.3% *m/z *of each other were deemed to correspond to one another and were 'retained' for comparative analysis of mean intensities. Peaks falling outside this criterion were considered to be unique to either pooled or individual spectra. For both sets ('retained' values only for set A) mean intensities for each peak cluster in the pooled and individual spectra sets were compared. This comparison was expressed as the median number (and interquartile range) of individual sample standard deviations (SDs) that pooled sample intensities lay from their respective individual sample means. This was done separately for cases and controls. In addition, for each peak cluster the ratios of the case mean intensity to control mean intensity in the individual samples were calculated and the deviation of this ratio for corresponding peaks in the pooled samples was expressed as the number of standard errors of the mean that pooled sample ratios lay from individual sample ratios [7]. The variation of the most significant biomarkers detected (p < 0.05, by Mann-Whitney U test) was also calculated.

## Results

**Figure 2:**
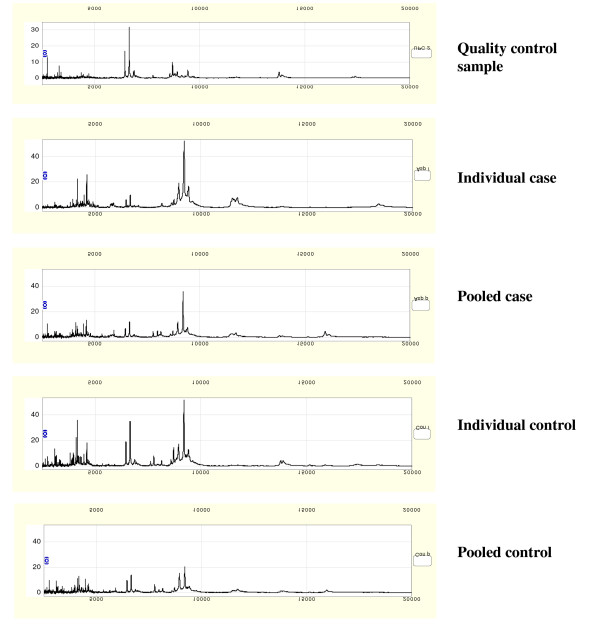
Representative SELDI spectra, illustrating a quality control spectrum, 'typical' individual case and control spectra and pooled sample spectra from the case and control groups. For each of the pooled sets, the 20 individual samples in each group were combined to form a pooled sample, from which a spectrum was generated under the same conditions as for individual samples.

**Figure 3:**
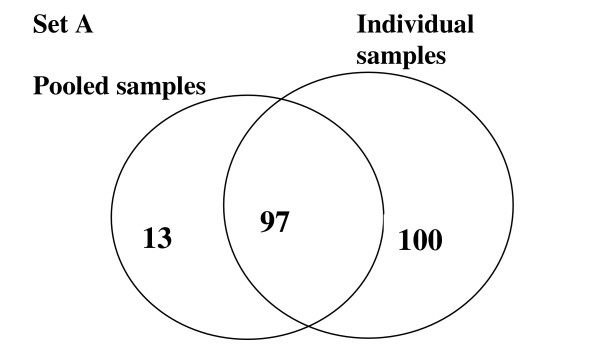
Numbers of peak clusters occurring in each dataset. Peak clustering for spectra in the pooled samples (Set A) was performed independently of clustering for the individual spectra. 97 peak clusters were common to both sets.

Representative spectra obtained from quality control, individual case and control and pooled case and control spectra are shown in figure 2. Where peaks in the pooled spectra were defined independently from peaks in the individual spectra (set A), 197 and 110 peak clusters were detected from the individual and pooled sample experiments respectively. 97 (49.2%) peak clusters fulfilled the pre-defined criterion of being within 0.3% of their *m/z *values between pooled and individual samples and were retained for comparative analysis (figure 3). Pooling resulted in the 'loss' of 100 peak clusters, while 13 peak clusters appeared to be unique to the pooled samples. In the quality control replicates, 44/55 (80%) of peaks with *m/z *> 2500 detected at a S/N ≥ 5 and a mass window of 0.3%, were present in all 7 replicates. Only a few clusters were generated across the mass range of 20,000 to 100,000 using a mass window of 0.3% and this hardly changed when using a mass window of 0.6% over the same range (data not shown).

Among these retained peak clusters, pooled-sample case and control intensities and case:control intensity ratios varied little from their respective values in individual samples. Of the pooled-sample case and control intensities, all lay <1 SD from their respective individual sample means, apart from 6/97 cases (which lay between 1 and 2 SDs) and 3/97 controls (which lay between 1 and 2 SDs). The case:control intensity ratios in the pooled samples were highly comparable to their corresponding ratios calculated from peak intensity means in the individual samples; all lay less than 1 SE from their values in the individual samples, apart from 1/97 which lay just over one SEs from its corresponding individual sample ratio). 59/97 (61%) of pooled sample case:control intensity ratios were less than 0.25 SEs from their respective individual sample intensity ratios.

**Figure 4:**
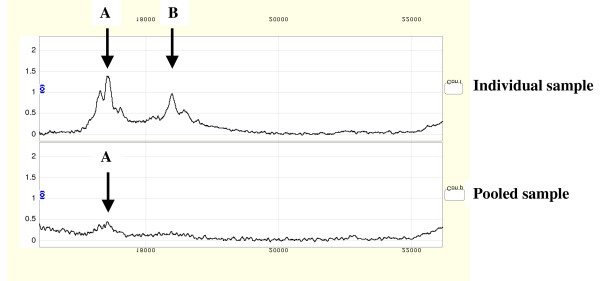
Illustrative individual and pooled spectra showing effects of pooling on peak intensity, resulting in diminished intensity of peak A and 'loss' of peak B to below the peak detection threshold.

Of the potentially discriminating peak clusters whose intensities differed statistically significantly between cases and controls, 21/35 (60%) were retained for analysis i.e. 14 were 'lost' as a result of pooling. Among these surviving clusters, a reduction in variation of intensities and ratios was observed (see figure 4 and Table 1). Pooled sample spectra were examined and potential peaks, falling within a 0.3% mass window of the individual sample clusters, but not identified by Biomarker wizard, were labeled manually for the 14/35 statistically significant peak clusters not retained for analysis. When these manually-labeled peak clusters were included in the data very little change in variation between pooled and individual samples was observed (see Table 1).

**Table 1:** Variation of Pooled Sample intensities from Individual Sample intensities, using independent clustering (A) or imposed clustering (B).

**Set, Significance level**	**Number of peak clusters in pooled samples retained for analysis**	**Median number of SDs pooled control cluster intensities are from mean individual sample intensities (IQR)**	**Median number of SDs pooled case cluster intensities are from mean individual sample intensities (IQR)**	**Median number of SEs pooled sample case to control ratios are from means of individual sample ratios (IQR)**	**Proportion of pooled sample case to control intensity ratios that lie less than 0.25 SEs from means of individual sample ratios**
A, all	97/197 (40.9%)	0.37 (0.18 – 0.61)	0.39 (0.16 – 0.62)	0.20 (0.10 – 0.33)	59/97 (61%)
A, p < 0.05	21/35 (60%)	0.29 (0.12 – 0.37)	0.33 (0.17 – 0.44)	0.16 (0.11 – 0.36)	13/22(62%)
A, p < 0.05 (manually added peaks, see text)	35/35 (100%)	0.34 (0.16 – 0.56)	0.33(0.18 – 0.57)	0.20 (0.12 – 0.45)	19/35 (54%)
B, all	-	0.40 (0.20 – 0.67)	0.43 (0.19 – 0.80)	0.21 (0.11 – 0.43)	109/197 (55%)
B, p < 0.05	-	0.30 (0.19 – 0.46)	0.40 (0.19 – 0.58)	0.14 (0.09 – 0.40)	23/35 (66%)

SD: standard deviation; SE: standard error; SDs and SEs are absolute in value, ignoring direction. IQR: inter-quartile range

**Figure 5:**
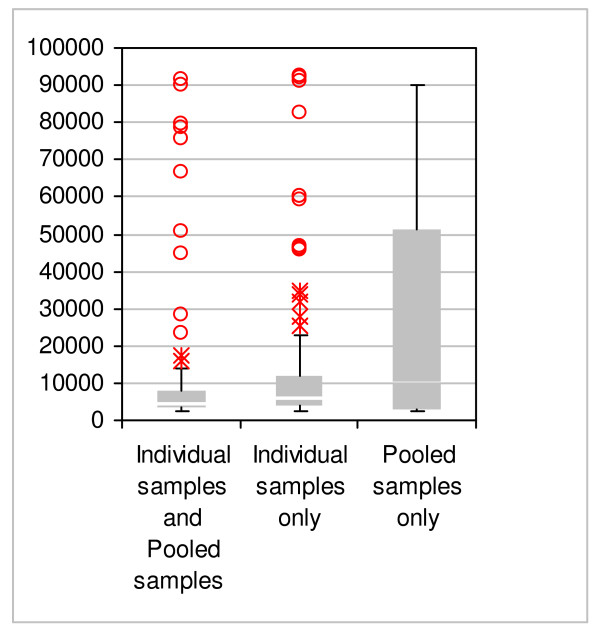
Distribution of *m/z *values for peak clusters found in both individual and pooled samples ("retained"), Individual samples only and Pooled samples only. Box = interquartile range, Bar = median; whiskers extend only as far as the furthest point within 1.5 interquartile ranges below the first quartile or above the third quartile. Asterix markers show outliers within 3 interquartile ranges above the third quartile, and circles show outliers beyond 3 interquartile ranges of the third quartile. Individual samples and pooled samples n = 100; Individual samples n = 97; Pooled samples only n = 13.

Factors associated with 'loss' of peak clusters as a result of pooling in set A were determined. Overall, median retained *m/z *values were lower than those not retained (4476 *vs *6163 p = 0.01). Of interest, the 13 peak clusters observed in pooled samples only and not in individual samples appeared to have higher *m/z *values (median *m/z *10165) than retained samples but the small numbers precluded statistical testing (see figure 5). Retention of peak clusters was more likely when they were found in a higher proportion of individual samples (median percentage representation in individual samples for retained *vs *non-retained clusters: 75% *vs *35%, p < 0.00001; 58% *vs *31.5%, p < 0.0004 in patients with peak intensities less than 1.0). Retention was also associated with higher peak intensities in both cases and controls, independently of whether the peak cluster was found in a higher proportion of individual samples. For retained *vs *not-retained, median (IQR) intensities were 2.55 (1.12 – 4.31) *vs *0.75 (0.42 – 1.60) and 2.61 (1.25 – 4.93) *vs *0.87 (0.50 – 1.41) for cases and controls respectively (p < 0.00001 for all comparisons). For peak clusters that were found in 50% or more individual samples, these differences were maintained (data not shown) indicating that low intensity peak clusters were lost despite being present in a significant proportion of samples.

Of the small number of statistically significant discriminatory peak clusters, there were no detectable differences in *m/z *values between retained and non-retained clusters (m/z 3451.2 *vs *5073 respectively p = 0.171) and again retention of peak clusters was much more likely if they were found in a higher proportion of individual samples (median percentage: 93% (65–98) *vs *35% (21–51); p < 0.00001 for retained *vs *non-retained for all sera). Retention of discriminatory peak clusters was also associated with higher peak intensities in both cases and controls. For retained *vs *non-retained, median (IQR) intensities were 2.0 (1.3 – 3.6) *vs *0.6 (0.2 – 1.1) and 3.0 (1.4 – 6.6) *vs *0.9 (0.2 – 1.2) for cases and controls respectively (p < 0.00001).

For set B, in which clusters generated from individual sample spectra were 'imposed' on pooled sample spectra, the same patterns were observed compared with set A with regard to case and control intensities. Imposed clusters appeared to increase variation of calculated pooled sample ratios (see Table 1).

## Discussion

This study set out to investigate the consistency of potential biomarker detection when individual case or control serum samples are pooled. Overall there was reasonable reproducibility of data between pooled and individual samples. Peak clusters that were consistently present in individual spectra and pooled spectra were of comparable intensities. Moreover, intensity ratios between cases and controls were largely preserved when measured in pooled spectra. However, a large proportion of peak clusters were lost after pooling. The majority of non-retained peak clusters were found only in a minority of individual samples and were generally associated with very low peak intensities. A plausible explanation for their loss would be dilution out during pooling, resulting in their falling below the noise threshold in the pooled sample. This 'averaging out' of what could be deemed to be outliers in the pooled sets may be useful, providing cases and controls are generally fairly homogeneous. On the other hand, it may obscure otherwise important differences between subsets of cases or controls that would be apparent on inspection of individual spectra at particular peak cluster values. This would be a significant limitation for the development of proteomic signature patterns. Importantly, our data suggest that low abundance proteins, even when represented in a majority of individual samples, may still be lost during pooling.

The appearance of 13 'new' peaks in pooled samples which were not present in the individual sample peak cluster set, is more difficult to explain. One possibility is that certain proteolytic interactions may occur in pooled samples but not individual samples, due to the presence of proteolytic enzymes in one or more individual samples. This might result in proteolytic cleavage of proteins in a pooled set, causing the disappearance of a 'parent' protein peak and the appearance of 2 or more cleavage product peaks. The frequency distributions of m/z values (figure 5) are not inconsistent with a few large proteins being lost in this manner generating a handful of new ones in pooled samples, although more work would be needed to demonstrate this. Proteolysis might be avoided by denaturing individual samples prior to pooling or by the inclusion of protease inhibitors, although this adds to the complexity of the sample pre-processing steps.

A further important limitation of pooling is that it precludes the development of multivariate classification models using machine-learning based algorithms such as support vector machines, artificial neural networks, decision tree classifiers etc. which require training of classifiers on individual spectra. Decision tree classifiers, in particular, are capable of accommodating considerable heterogeneity between individual spectra that nevertheless belong to the same diagnostic category. It is well recognised that a high degree of inter-individual proteomic variability is seen in SELDI serum spectra and that multivariate classifiers are a powerful method for assigning spectra to broad diagnostic groups [8,9]. Pooling will result inevitably in the loss of individual spectral information.

## Conclusion

Taken together, these data suggest that pooling serum samples may be associated with significant loss of potential biomarkers using SELDI-ToF MS, despite there being low variability in intensity for potential biomarkers that are retained. Concerns exist about the diluting out and loss of low abundance proteins found in select subsets of cases and controls. One area in which pooling might be useful is in the initial selection of chip surface on which to profile sera. While in ideal circumstances, the most comprehensive profile might be achieved by running all samples on all available chromatographic surfaces, typical studies involve the analysis of many hundreds of samples and cost considerations often preclude such an approach. The profiling of pooled samples may provide a more reliable method for the rapid screening of multiple chromatographic surfaces for potentially discriminating profiles than merely comparing chip surfaces on the basis of 5–10 individual samples, as is currently common practice. However, further work, investigating the effects of pooling on biomarker detection, is warranted and until then, pooling samples is not a preferred strategy for biomarker detection.

## Competing interests

The authors declare that they have no competing interests.

## Authors' contributions

STS and DA jointly conceived the study, performed the analysis and co-wrote the paper. DA performed the SELDI-ToF experiments.
